# Fibre Bragg Grating Based Acoustic Emission Measurement System for Structural Health Monitoring Applications

**DOI:** 10.3390/ma14040897

**Published:** 2021-02-13

**Authors:** Sagar Jinachandran, Ginu Rajan

**Affiliations:** 1 School of Electrical, Computer and Telecommunications Engineering, University of Wollongong, Wollongong, New South Wales 2522, Australia; sj317@uowmail.edu.au; 2 ARC Training Centre for Automated Manufacture of Advanced Composites, UNSW, Sydney, New South Wales 2052, Australia

**Keywords:** optical fibre sensors, acoustic emission, fibre bragg gratings, structural health monitoring

## Abstract

Fiber Bragg grating (FBG)-based acoustic emission (AE) detection and monitoring is considered as a potential and emerging technology for structural health monitoring (SHM) applications. In this paper, an overview of the FBG-based AE monitoring system is presented, and various technologies and methods used for FBG AE interrogation systems are reviewed and discussed. Various commercial FBG AE sensing systems, SHM applications of FBG AE monitoring, and market potential and recent trends are also discussed.

## 1. Introduction

It is known that civil, aerospace, marine, pipeline, and mechanical infrastructures deteriorate after being built due to ageing induced cracks, man-made hazards such as vehicle collisions and blasts, and natural disasters such as earthquakes or hurricanes [1,2,3]. These can have deleterious effects on the integrity of the engineering structures during their service life, leading to catastrophic operational failures. It is important to establish procedures to assess the deterioration of the structures which are strategically important to human life and safety over their serviceable life to facilitate the maintenance and/or rehabilitation planning processes in this modern society that encourages sustainable development. Hence, structural health monitoring (SHM) which can help detect, locate, and diagnose damages and predict their prognosis is of paramount importance. Some of the recent studies conducted by industries show that early detection of damages can save great expenses and increase the lifespan of a structure, thereby providing value to the industry and society in general [4]. Therefore, a technologically and economically feasible SHM method to identify and assess defects/cracks in structures at an early stage of formation to the micro/nano scale is essential to safeguard the structural integrity and operation throughout the service life.

There are several technologies being used for SHM currently, such as the elastic-wave-based method, acoustic emission method, electromechanical impedance method, vibration method, strain sensitivity measurement based method and many others that can determine the location, severity, and extent of damages [5]. Non-destructive evaluation (NDE) is emerging as a key player in SHM due to its ability to correlate the measured physical parameters to quantitative information about anomalies [6]. Among the different techniques of NDE to detect the defects in the structure, the acoustic emission (AE) measurement technique is well-known, which detects stress waves generated by defects in materials thereby allowing continuous and real-time structural monitoring. AE refers to the generation of transient elastic waves during the rapid release of energy from localized sources within a material, which occurs due to the initiation and/or propagation of cracks in metals, surface degradation, including corrosion and delamination and matrix cracking in the case of composite materials, and these waves can travel through all materials except vacuum [7].

**Figure 1:**
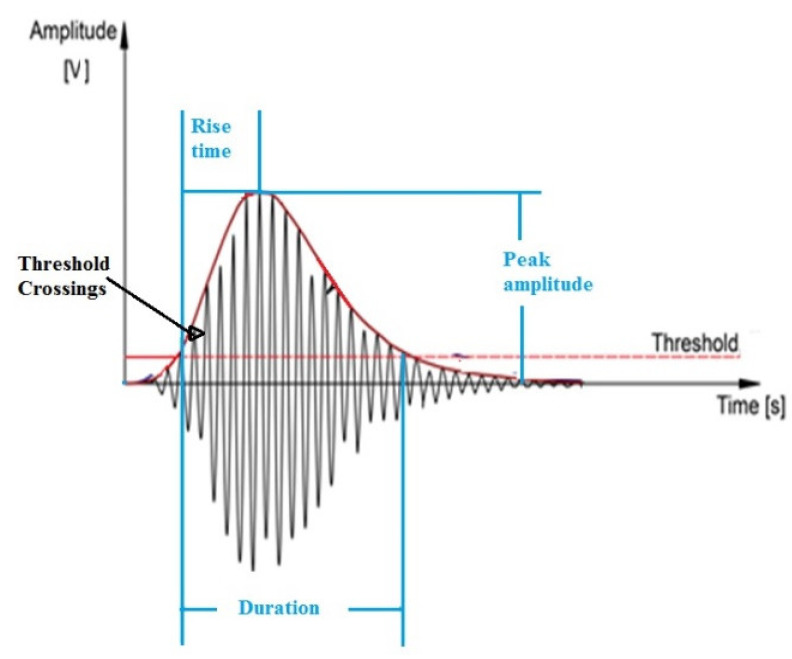
A typical acoustic emission (AE) waveform.

A typical AE waveform is shown in Figure 1, which is generally a mixture of signal and noise [8]. The amplitude of the AE represents the magnitude of the event, and rise time is the interval between the time a signal is set off and the time when it reaches the maximum amplitude. AE duration and energy will also provide crucial information about an event. AE duration is the time taken from the triggering of the signal to the time the signal falls below the threshold value, while energy is measured as the area under the rise time and above the threshold. Any signal which goes above the threshold value is considered towards an AE count. 

Current NDE technologies to measure AE involve sensors based on piezoelectric ceramics, especially Lead Zirconate-Titanate transducer (PZT) systems, field-programmable gate arrays, and the Fabry–Perot interferometer [9,10,11]. Though PZT-based systems are the most common ones, they are not quite conducive in monitoring micro-crack activities, are unable to determine cracks deep inside the structures, have a tendency to debond or fracture under large stress and are susceptible to electromagnetic interference (EMI). PZT sensors often require preamplifiers and signal conditioning units, and their size is not suitable for being embedded into a material without affecting the material integrity. They also have limitations to be used in harsh environments like at high temperatures or high-voltage environments and also have a low power handling capacity [12,13,14,15].Other traditional NDE techniques include ultrasonic scan, the eddy-current method, radiography, and passive thermography [16]. These NDE techniques are effective in detecting damages to materials and structures but cannot monitor the micro crack nucleation and propagation. Therefore, there is a great need to implement advanced and new types of sensors that can be integrated with structures, such as the ones based on optical fibres, allowing the in-situ monitoring of structures for micro-cracking activities that are precursors to a major failure.

Optical fibre sensors (OFS) are considered as an excellent technology with the highest potential for continuous real-time monitoring owing to their accuracy in carrying information, invulnerability to different types of interferences, reliability and versatility, small size and lightweight, high bandwidth, robustness and resistance against harsh environments, high sensitivity, ability to sense several parameters (strain, pressure, corrosion, temperature, and acoustic signals), chemical inertness, biocompatibility, and ability to be integrated into the system [17,18,19].OFSs have their applications in various fields such as SHM, seismic monitoring, transformer partial discharge diagnosis, underwater acoustic monitoring, aerospace safety, airborne acoustic detection, photoacoustic spectroscopy, photoacoustic imaging and other such fields [20,21]. There are different kinds of AE OFS technologies such as Fabry–Perot (F–P) cavity sensors, fibre optic ring sensor, fibre optic coupler AE sensors, and fibre Bragg grating (FBG) AE sensors [22,23,24,25,26,27,28,29,30] which have their own advantages and disadvantages. Among the different types of OFS’s, FBGs are widely used and considered the most popular technology for structural health monitoring systems [31]. Furthermore, their multiplexing capability offers the possibility to dramatically reduce the cumbersome wiring requirements of traditional sensors [17]. There are still many concerns to be deciphered for developing a compact, accurate, stable, and economical FBG AE interrogation system for large scale commercial engineering applications. This review provides the current landscape of the various FBG-based technologies for AE detection and its advantages and pitfalls.

## 2. Fibre Bragg Gratings and Interrogation Systems

**Figure 2:**
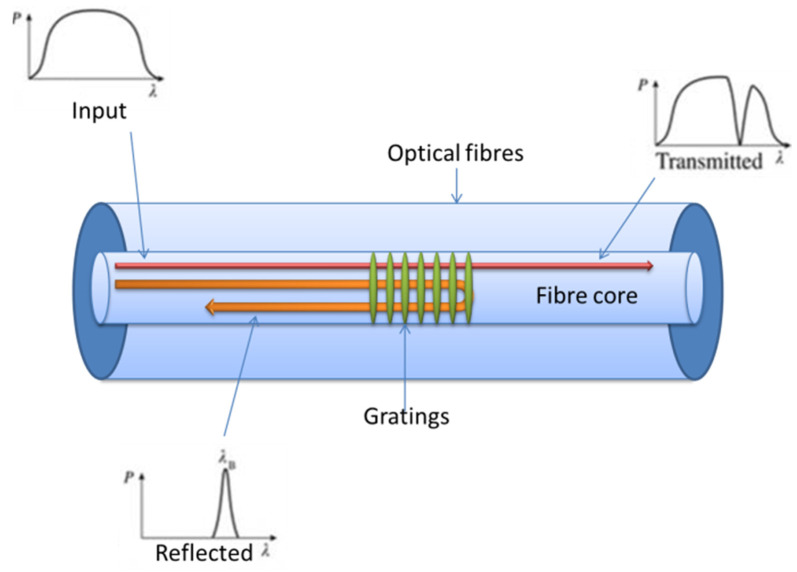
Schematic of a fibre Bragg grating (FBG) and its operating principle.

FBGs are the most commonly employed fibre optic sensors in SHM applications since their instigation in 1978 [32,33,34]. FBG in its most basic form comprises a short section of single-mode optical fibre, in which the core refractive index is modulated periodically using an intense optical interference pattern [35], typically at UV wavelengths. This periodic index-modulated structure enables light to be coupled from the forward propagating core mode into a backward propagating core mode generating a reflection response. The peak reflected Bragg wavelength λ_B_ is given by the following:

(1) $$\lambda_{B}=2n_{\mathrm{eff}}\Lambda$$

where *Λ* is the grating period and *n_eff_* is the effective refractive index of the fibre. The basic principle of an FBG is illustrated in Figure 2. The peak reflected Bragg wavelength is sensitive to a range of physical parameters, such as strain, pressure, temperature, and other external parameters.

The AE measurement capability of an FBG is correlated with the strain sensitivity where the Bragg wavelength exhibits a change due to the change in the grating pitch and refractive index via the strain optic effect, when an external strain is applied. The strain induced wavelength shift is given as follows:

(2) $$\Delta \lambda_{B}=\lambda_{B}\left(1-\rho_{\propto }\right)\Delta \varepsilon$$

where $\rho_{\propto }$ is the photo-elastic coefficient of the fibre and Δ*ε* is the induced strain. The photo-elastic coefficient can be given by the equation,

(3) $$\rho_{\propto }=\frac{n_{eff}^{2}}{2}\left[\rho_{12}-v\left(\rho_{11}-\rho_{12\;}\right)\right]$$

where $\;\rho_{11}$ and $\rho_{12}$ are the coefficients of the strain-optic tensor of the optical fibre and *ν* is the Poisson ratio. 

When an acoustic wave impinges on an optical fibre with an FBG, the refractive index of the fibre and the FBG period are modulated by the acoustic wave-induced mechanical strain in the fibre through the elasto-optic effect. The dynamic wavelength shift, corresponding to the acoustic signal-induced strain Δ*ε* (*z*, *t*), can be written as follows:

(4) $$\lambda_{B}\left(z,t\right)=\lambda_{B}\left(1-\rho_{\alpha }\right)\Delta \varepsilon \left(z,t\right)$$

where Δ*ε* (*z*, *t*) is the induced dynamic strain and *z* is the axial direction along which the longitudinal strain propagates, and t is the time. In a simple case, a Gaussian strain field generated by the AE wave along the FBG axis can be represented as follows [36]:

(5) $$\varepsilon \left(z,t\right)=\;\varepsilon_{m}\cdot e^{\frac{-(t-t_{o)}}{a^{2}}}sin\left(\frac{2\pi }{\lambda }z-2\pi f\right),\;\;\;\;\;\;z\in \left(0,l\right)$$

where *ɛ_m_* is the strain field displacement amplitude, *t*_0_ is the arriving time, *λ* and *f* denote the wavelength and frequency of AE waves respectively, and *l* is the FBG gauge length.

The interrogation system for FBGs plays an important role in measurand parameters such as sensitivity and range as it extracts the measurand information from the optical signals collected from the sensor heads. This information is typically encoded in the Bragg wavelength shift; therefore, interrogators are typically used to create a readout of the wavelength shift and provide measurand data [37]. The general requirements for an ideal interrogation method are high resolution and accuracy with a large measurement range, compatibility for multiplexing, high measurement speed, and cost-effectiveness. Typically, a wavelength shift detection resolution ranging from sub-pico meters (<10 pm) is required for most applications. A wide wavelength range (tens of nanometers) is required when interrogating multiple FBGs that have different Bragg wavelengths and should be able to cope with multiplexing topologies, such as wavelength division multiplexing. The cost of an interrogation system should also be competitive with those of other optical sensors or conventional electrical sensors [38].

## 3. FBG Interrogation Systems for AE Monitoring

In order to measure an AE-induced strain wave, the main hurdles to be overcome are high data acquisition rate and sensitivity that is needed to recreate the AE signal and the amount of extraneous noise that the sensor senses through vibration and other interferences. The conventional FBG interrogation system is limited by its measurement frequency range of operation (typically in the range of 1–10 kHz) and sensitivity that would limit the system to measure AE signals. An ideal AE interrogation system should be one that can at best be able to work in a frequency range of 1–500 kHz and should have the capability to detect pico and femto strains as the AE induced strains from micro and nano-cracks/defects in structures would be in this range [39]. 

There are several approaches to effectively measure AE events using an FBG-based system. Most FBG interrogation systems for detecting an AE signal operates on the principle of converting the dynamic shift in reflected Bragg wavelength into intensity variation of the output signal using an optical filter. In other methods, the dynamic shift in the reflected Bragg wavelength is converted to a change in phase of the output signal, where the dynamic shift in the reflected Bragg wavelength is converted to a change in phase of the output signal. In either of these methods, the acoustic emission signal induced shift in the Bragg wavelength corresponds to either intensity or phase shift of the output wavelength and can be intercepted using signal processing systems to detect the defect in the structure [40]. Based on the operating principle we have classified the technologies into major groups as listed below.

### 3.1. Power Detection and Edge Filter Detection Methods

**Figure 3:**
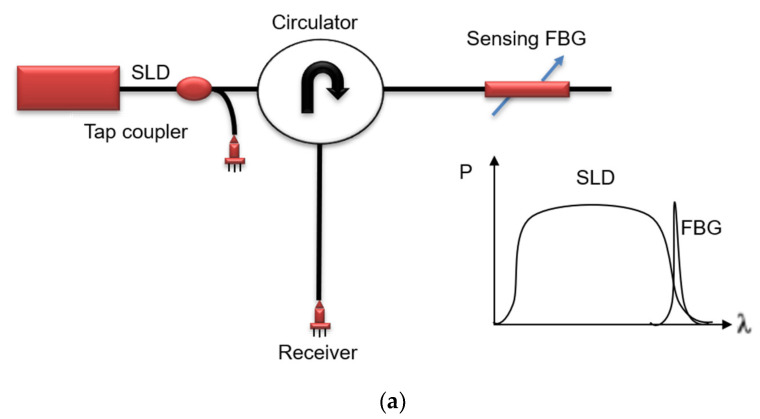

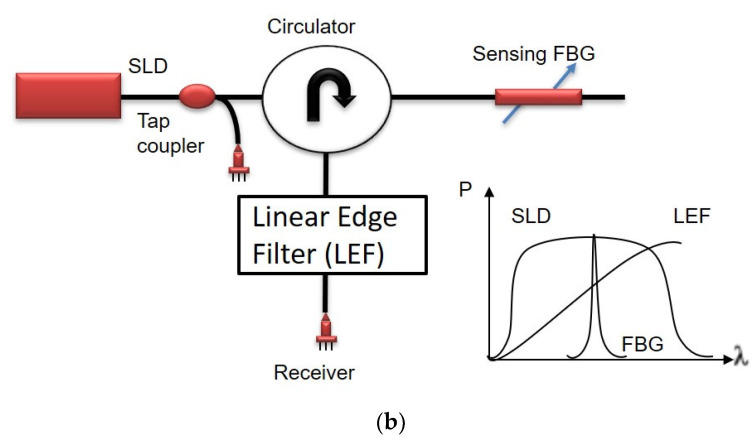
Optical circuits of (**a**) power detection method (**b**) edge filter method.

In the power detection [41] method, a broadband source is used to illuminate an FBG and a filter is used to demodulate the Bragg wavelength shift to power intensity shift. This method has multiplexing ability, but it has a relatively low sensitivity because of the spectral profile of the broadband light source. On the other hand, in the edge filter technique [42,43,44] a tunable laser source (TLS) or a broadband source is used to adjust the source wavelength to the linear region of the FBG signal spectrum. In this method, a spectrally dependent filter is used to detect any dynamic shifts in the FBG wavelength spectrum and converted to intensity variation by the wavelength-dependent filter. The sensitivity is relatively high for edge filter detection compared to power detection method as the noise level in the TLS is lower than that of the broadband laser source as the spectrally-dependent source which is measured towards the end of the spectrum which results in change of intensity at the detector, however for an edge filter detection method the FBGs are selected such that Bragg Wavelength is at the 3 dB point of absorption transmittance and the light not transmitted to the detector is absorbed by the filter. A number of different edge filters are reportedly used, such as a matched FBG [29], a linear edge absorption filter, an interference filter [45], a WDM coupler [46], an arrayed-wave guide grating (AWG) [47], and a dense wavelength division multiplexing (DWDM) filter. Optical circuits of the edge filter and power detection methods are illustrated in Figure 3a,b respectively.

### 3.2. AE Detection Using an Optical Fibre Based F-P Sensor and Quadrature Recombination Technique

**Figure 4:**
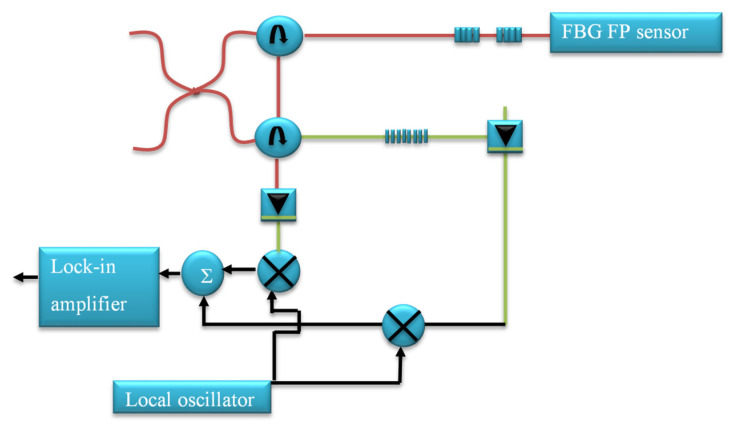
Fabry–Perot and quadrature recombination technique for AE monitoring [48].

In this method, AE detection is carried out using an FBG-based Fabry–Perot (FBG-FP) sensor and the quadrature recombination technique [48,49]. The working principle of this system is to combine and launch two optical signals with different wavelengths through a 3 dB coupler in a FBG-FP cavity as shown in Figure 4. In this approach the laser wavelengths are set in quadrature with one of the lasers tuned to the most sensitive region of the spectrum of the cavity and the other laser tuned to the least sensitive region. The reflected signal from the two lasers gathered from the photo detector is processed and recombined employing a signal processing system. As the wavelengths of the laser are set in quadrature, the AE sensitivity is improved, and the system is capable for high frequency measurement. But the disadvantage is that the lasers should be tuned manually to the quadrature point and further research is required make it the system more compact for practical applications.

### 3.3. FBG AE Sensors by Altering the Gratings and Physical Configuration

The principle behind this ‘modified FBG sensor’ is the relation between the spectral response of the FBG and the ratio of the grating length to the AE signal wavelength. If the wavelength of the AE signal becomes similar to the grating length, the FBG spectrum can be highly distorted because the grating will be subjected to a different internal strain gradient lengthwise. Typically, shorter length FBGs are chosen as it enhances the sensitivity for AE-induced strain. An AE sensor with wider temperature and strain sensitivity, capable of pure AE detection and having high mobility has been designed and experimentally demonstrated by J. Lee et al. [50]. FBG-based AE interrogation system used in this study consists of a narrow band laser source emitted from a tunable laser diode (TLD). The size of the FBG used was 1 mm in length and 125 μm in diameter. The pitch of the FBG is modulated by the AE generated and the reflected intensity variation is detected by the photodiode.

To enhance the sensitivity of AE measurements, FBG sensor heads are modified by changing the bonding configuration (to the substrate), and are packaged and coated with different materials. One example is found in a comparison study where two types of sensor heads were tested where one was fully bonded to the sensing element and the other was only partially glued at one end only and the other end was lying free, acting like a cantilever. It is shown that a cantilever-type sensor has high resonance frequency and greater sensitivity [51].It is also reported that the sensitivity of FBGs for pressure and acoustic detection can be enhanced by coating the grating region with a material of lower elastic modulus than the optical fibre [52]. For a given acoustic pressure, the role of FBG coating is to enhance the dynamic strain experienced by the sensor by a factor given by the ratio between the fibre and the coating elastic modulus. According to this, a suitable coating can tailor the sensor directivity, the bandwidth, and the acoustic sensitivity [53]. Sensitivity can also be improved by different packaging techniques [54] and by reducing the fibre diameter. A summary of the effect of the physical configuration of the sensors on the AE measurement is presented in Table 1.

### 3.4. Spectrometric FBG AE and Ultrasonic Interrogation Systems

**Figure 5:**
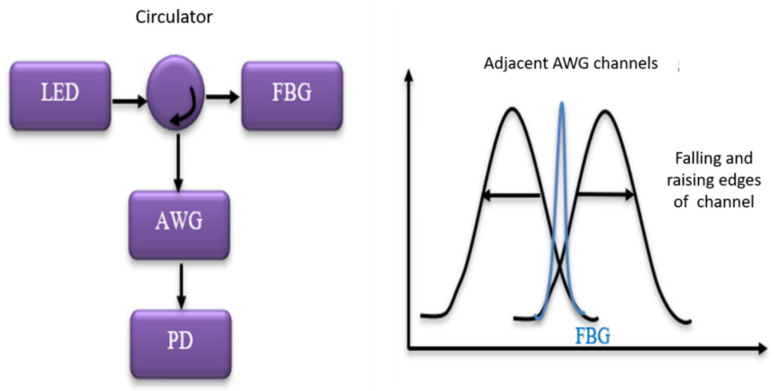
FBG interrogator system using an arrayed waveguide grating (AWG) [55].

One of the major challenges when dealing with the AE signal is that the interrogation speed should be in the scale of hundreds of kHz to sense an AE signal. In recent years, high speed wavelength interrogation techniques have been developed with a data acquisition rate over 100 kHz, such as a Fourier-domain mode-locked laser, wavelength-tunable mode-locked laser, and wavelength-swept laser based systems which are bulky, costly and highly complex. For frequencies below 1 kHz, wavelength sweeping by mechanical moving parts, such as with a tunable laser system (TLS) and the F–P filter type of wavelength interrogation techniques, are typically used. For frequencies above 1 kHz, a filter without mechanical moving parts is used for wavelength interrogation. In spectrometric interrogation methods for AE measurements, configuration based on matching FBG, a long period fibre grating, a laser diode, and an AWG have been used [40,41,42,43]. Schematics of the FBG interrogation system using a laser diode and an AWG is shown in Figure 5 [55]. In this configuration, the wavelength of the laser diode is first set to a certain position of the FBG spectrum for the measurement of AE signal as shown in Figure 5. Measured reflected signal power intensity at the photodetector is altered depending on the shifts in the FBG spectrum and thus correlating the wavelength change to an intensity change. In this configuration, the AWG acts as a wavelength dependent optical filter which has two channels, a rising edge and a falling edge. For AE measurement, the Bragg wavelength is set to the middle of the two channels. Therefore, if there is any shift in the FBG reflectivity spectrum, it will reflect as a change in the intensity of the reflected signal [42]. A wavelength interrogation system based on an Echelle diffractive grating (EDG) similar to AWG, based on planar light wave circuits technology has also been demonstrated for AE monitoring and was able to achieve the wavelength resolution of less than 1 pm with a measurement accuracy of ±10 pm [56].

### 3.5. Bragg Grating-Based Laser Sensor System with Interferometric Interrogation and WDM

**Figure 6:**
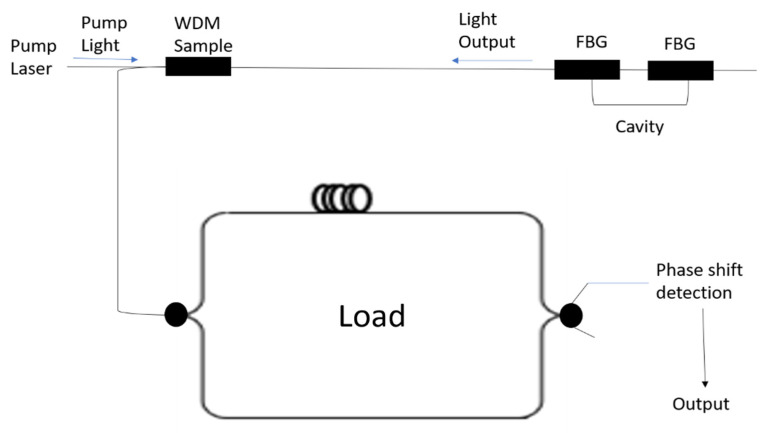
Interferometric Interrogation along with WDM [57].

The potential of FBGs to be used as spectrally narrow band reflectors for creating in-fibre cavities for fiber lasers has been utilized in this approach. As an example, the use of FBG-based lasers as sensors was described by some authors [57]. An interferometric detection technique is demonstrated for interrogating laser wavelength shifts due to measurand-induced laser cavity strain for ultrasonic waves with high resolution. The FBG laser sensor (FBGLS) is an optical fibre doped with Erbium of a finite length with two optically matched FBG on either ends that acts as the cavity for lasing. The light from the pump laser is diverted through a WDM coupler to the interferometric interrogation system as shown in Figure 6. The laser source is connected through the WDM coupler to a Mach-Zehnder interferometer (MZI) and any shift in wavelength is converted to interference pattern phase shifts [57] and was recorded by a dynamic signal analyser. The phase modulation is determined by the product of the laser cavity strain and the imbalance in the path of the interferometer. For a single-mode FBGLS, the interferometer phase shift is dependent on the output wavelength (or optical frequency) and the interferometer optical path difference (OPD). The line width of the laser emitted is very narrow with increased coherence length and so a large OPD can increase the sensitivity of the system. The interferometer phase noise was translated to a FBGLS strain resolution of 5.6 × 10^−14^/ √Hz. The strain resolution obtained is due to the limit imposed by the thermally induced cavity length fluctuations of the lasers.

### 3.6. Intensity Demodulation Fibre Ring Laser Sensor System for AE Detection

The commonly used FBG interrogation system uses a narrowband laser source whose spectrum is tuned according to the central wavelength of the linear region in the reflected spectrum from the FBG, thus performing an intensity-based demodulation. The sensitivity of the system depends on the wide spectral slope of the FBG. As explained in the previous sections, detection sensitivity also becomes unstable when the AE signal wavelength becomes comparable to the grating wavelength. Having a narrow bandwidth tunable laser source also increases the cost incurred for the system; therefore, a fibre ring laser (FRL) with a narrow bandwidth tunable optical band-pass filter (TOBPF) was developed for AE detection [58]. The system contains a conventional FBG sensor as the sensing element in the cavity of the FRL, which modulates the intensity in accordance with the presence of an AE signal. The extremely narrow bandwidth of TOBPF ensures that the FRL strictly sources a frequency in the linear region of the reflected spectrum of the FBG. The key benefit of this approach is a highly sensitive tool to capture AE signals at low cost.

**Figure 7:**
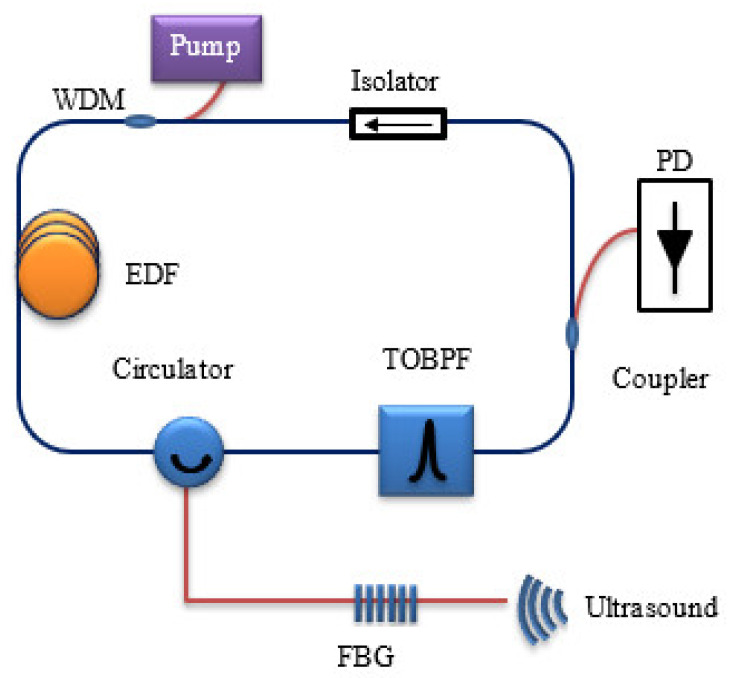
FBG Interrogation using a fibre ring laser (FRL)sensor for AE Detection [58].

As shown in the Figure 7, the FRL consists of an Erbium doped fibre (EDF) that is fed by a laser diode along with an optical fibre isolator to ensure that unidirectional lasing occurs in the system. When an AE is detected by a conventional FBG, which causes the reflected spectral wavelength to shift, the TOBPF in the lasing cavity loop ensures that the laser source transmitted spectrum is so narrow that it falls exactly on the linear region of the FBG reflection spectrum. In accordance to the strength of the AE signal, a spectral shift is observed in the FBG.

Furthermore, due to the highly aligned laser spectrum, the effect of the ultrasonic signal causes a modulation of the loss of the cold cavity of the FRL and the output optical power of the FRL varies according to the loss modulation. A photodetector placed in the loop can detect the variation in output power from the laser, which, in turn, detects the presence of an AE signal. This proposed idea has theoretically been proven to be effective in detecting the presence of a high-frequency ultrasonic signal as well as low-frequency strain applied on a structure [58].

## 4. Commercial FBG AE Interrogation Systems

Commercially available FBG AE interrogation systems are evolving from benchtop laboratory instruments that were too cumbersome and heavy to be permanently installed in an operational platform to field deployment models with rugged design. Now there are several commercially available FBG-based AE interrogators few of which are summarized in Table 2 [59,60,61,62,63]. The AE frequency range of these devices range from 20 kHz to 3 MHz, with a resolution ranging from 0.1 pm to 5 pm and accuracy from 2 pm to 5 pm. Several emerging applications of FBG sensors where dynamic/AE strain sensing is required is prompting many industries to improve their device specifications to compete with other traditional systems. A comparison between the proposed FBG AE interrogation system is also shown in Table 3.

## 5. Application of FBG-Based AE Monitoring System and Its Market Potential

The most common applications of FBG AE systems are in SHM for smart structures to monitor initiation and propagation of cracks and micro fractures. FBG-based AE detection has been successfully demonstrated in a wide range of applications such as SHM in the civil, aerospace, and marine engineering, human biometric health monitoring in the medical industry; and several other applications [64,65,66,67].

In the aerospace sector, the rise in demand is pushing aircrafts to be used beyond their service life and hence there is an increasing need and effort to be put in inspection and maintenance to ensure the safety of the operating aircraft [65]. Research has proven that monitoring of AE can be a low-cost and effective solution to this problem. The simplicity with which these sensors can be incorporated in both existing and new structures can increase the demand for this technology. The routine health inspections for these structures involve not only NDE but also disassembling and re-assembling, which may cause more damage to the parts of the vessel. Paving the foundation for next-generation technology, smart sensing technology can completely eliminate the conventional and complex cost associated with these routine inspections and replace them with real-time on board monitoring to improve operational life cycle, reliability and safety [68]. Recently, shape memory alloy (SMA) wires were integrated into composite structures to create “smart composites” with the ability to control the shape and mechanical properties of composite structure. FBGs embedded into an SMA-based composite are highly reliable sensors as they are able to provide static and dynamic strain information on the SMA actuators operation [69]. Detection of impact damage either due to birds strike, hail or during ground service operations are also of great importance to the aerospace sector. Surface mounted FBGs and high speed interrogation systems were used to monitor this impact damage on aircraft.

In marine engineering and marine applications, the effect of cavitation erosion has been the main challenge to structures. The cost incurred to these industries due to these cavitation erosions are to the amount of millions across the world [69]. Due to the damage inflicted by vortices, caused by cavitation, microbubbles build up gradually. The microcavity, which produces ultrasonic shock waves, in turn erodes the solid material from the marine structure. These acoustic waves of high frequency are related to crack formation and propagation through the surface of the material. Therefore, effective AE detection and monitoring systems are required to prevent cavitation erosion.

There has been tremendous growth in research and development in the field of FBG-based AE monitoring in SHM for civil engineering applications including buildings, piles, bridges, pipelines, tunnels, and dams. Civil infrastructures with embedded FBGs can predict structural failures. In order to protect buildings and structures from shocks which results from earthquakes piles are used, 30 FBG sensors are multiplexed into arrays which contain 6 optical fibres for monitoring in a marine pile which were 60 ft in height and had a diameter of 2 ft. Among which 4 FBGs were used for monitoring strain and two were used for monitoring temperatures. The FBGs were monitored using a commercial FBG interrogation system based on Fabry–Perot filter technique [70]. Fibre reinforced plastics (FRP) and smart composites can be used in the construction phase helping in real time monitoring. Safe monitoring of construction and maintenance of railway tunnels is done using FBGs. Another example of testing of pile foundation was done in the new factory in Taiwan, where piles of diameter 1.2 m and length of 35 m were used and nine 4 m long gauge OFS were used to test compression and pull out on the pile and strain and load eccentricity. Sixteen 4 m long gauge optical fiber sensors (LGFOS) were installed to monitor the average curvature, Young’s modulus, longitudinal strain, vertical displacement, forces on the piles, the properties of soil, critical strain when cracks occurs on the pile, loading capacity of the pile and failure mode [71].

Another application of FBG AE system is in road traffic monitoring. A dynamic traffic testing system was developed by Udd et al. who used 28 specially designed FBG traffic sensors among which 26 survived. These sensors have a 0.1 micro-strain resolution with a400 micro-strain at 10 kHz sampling rate which can satisfy traffic monitoring requirements [72]. Another example of the traffic monitoring using FBGs were implemented in I-84 freeway to test the sensors and the counter which were ongoing for half a year which can easily discriminate tractors, buses, and trailers and these can also be used for determining the traffic in adjacent lanes as well. The signal’s amplitude is closely proportional to the weight of vehicle, speed of a vehicle and the direction of the driving vehicles.

**Figure 8:**
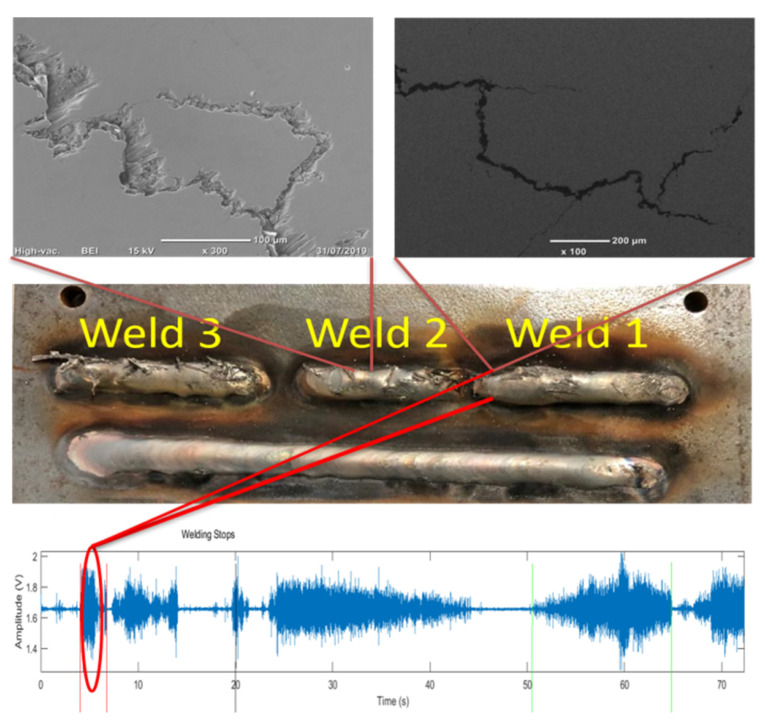
Confirmation of cold cracks from SEM imaging and its correlation with time domain response [73,74].

There have been high temperature environment applications for FBG AE sensing namely in welding for the detection of hydrogen induced cold cracks [66,73,74] where the FBGs can effectively detect and locate the formation and propagation of these cracks. The detection is carried out by using multiple packaged FBG sensors. The time domain response and the frequency response of the cold cracks were observed using the interrogation system and using the source localization algorithm the cracks were located and were confirmed using SEM analysis as shown in Figure 8.

As seen above FBG is a low cost sensor with the ability to sense various parameters like strain, temperature, pressure and acoustic emissions which, along with useful characteristics like multiplexing ability make them supreme in being implemented in a variety of applications in various fields and industries. The FBG market is composed of sensors, interrogation systems and services. The combined present global market size of the FBG sensors and arrays segment is estimated to be in the range of $15M to $35M USD a year, with an annual growth rate of 15% to 25% [75]. Market surveys indicate that at 25.3% compound annual growth rate, FBG market size is expected to reach 2070 million USD in 2023 compared to 670 million USD in 2020. With proper packaging and publicizing techniques FBG sensing and interrogation industry will grow tremendously.

## 6. Conclusions

The research evaluates the current conventional FBG interrogation systems and points out the limitations in terms of their application and implementation in AE. Upon advancement in technology, the rise in demand of industrial standards has necessitated a periodic structural monitoring for various industrial facilities. AE has proven to be an effective indicator and monitoring tool for SHM. Replacing the conventional PZT sensors with optical fiber sensors have proven to be more efficient in terms of the following: sensitivity enhancement of the system; ruggedness of the system; the possibility of multiplexing in sensing systems; enhancing the longevity of the sensing system installed; speed of capture/detection of an event; immunity to noise/capability to filter out noise as an inherent nature of the system. The current cost associated with the FBG sensing system is higher than the conventional system, but, with future research in the field of fiber optic sensing and wider application, this cost is anticipated to reduce exponentially. Therefore, a commercially cost-effective option should be rolled out soon, broadening the scope of its application in various fields of engineering.

## Figures and Tables

**Table 1:** Effect on physical configuration of the sensor on sensitivity.

Modification	Sensitivity
Fully bonded	Less
Cantilever type	Higher resonance frequency
Coating material of less elastic modulus	Higher can tailor directivity and bandwidth
Magnetic packaging	Enhances
Reduced diameter	Enhances

**Table 2:** The commercial FBG AE interrogators and their specification [59,60,61,62,63].

Manufacturer	Model	Frequency Property(kHz)	Resolution Property	Wavelength Accuracy	Application
**Ibsen Photonics A/S**	I-MON 256 HS	35(Measurement frequency)	<0.5 pm	5 pm	Impact
**Smartfibres Inc.**	Smartscan	25 (Scan frequency)		<5 pm	Impact
**Redondo Optics, Inc.**	FBG-TransceiverTM-500	20 (Sampling rate)	5 pm	5 pm	Impact
	FAEsense	585 (AE frequency)	0.1 με/Hz		Ultrasonic
**Intelligent Fiber Optic Systems corporation**	I*Sense^®^ HS48M	Maximal 3000 (detection speed)	0.1 pm	2 pm	Ultrasonic

**Table 3:** Comparison between proposed FBG AE interrogation techniques [35,36,38,39,40,41].

	Wavelength Interrogation Method	F–P Sensor with Quadrature Recombination	Interferometric and WDM Interrogation Approach	Intensity Demodulation Using FRL
Application	- SHM in aerospace - Operational load and damage detection	- Failure detection of structures in aerospace industry - SHM in composite structures	- Detection of low-frequency impact/impact test - Detection of acoustic range signals	- Application extended to SHM for composite structures, aerospace, marine etc.
Multiplexing capability	- WDM	- Not experimentally demonstrated	- WDM	
Detectable sensitivity	1 με	-	7 × 10^−15^ε/√Hz	−
AE frequency measurement	Up to 500 kHz	Up to 600 kHz	Tested for up to 10 kHz	Suitable for < 400 kHz
Advantages	- Easy to multiplex multiple sensors - Robust and low cost - Easy to install on structures	- Capable of compensating for environment perturbation - Compatible with different fiber optic sensors	- Very fast response - Highly sensitive real-time system	- TOBPF compensates for unwanted low-frequency wavelength shifts - Robust and simple setup - Potentially low cost
Disadvantages	- Trade-off between sensitivity and accuracy	- Difficult in tuning the two lasers for initial setup - Not cost effective	- Capable of detecting only low-frequency acoustic signals	- Not suitable for AE with low-frequency range

## Data Availability

All data included in this study are available upon request by contact with the corresponding author.
